# Adherence and quality of life assessment in patients with asthma treatment with budesonide/formoterol via the Elpenhaler device: the COMPLETE study

**DOI:** 10.1186/s12890-022-02049-0

**Published:** 2022-06-27

**Authors:** Konstantinos P. Exarchos, Nikoletta Rovina, George Krommidas, Dimitrios Latsios, Athena Gogali, Konstantinos Kostikas

**Affiliations:** 1 Respiratory Medicine Department, University of Ioannina School of Medicine, Ioannina, Greece; 2 1St Department of Respiratory Medicine, Medical School, National and Kapodistrian University of Athens and “Sotiria” Chest Disease Hospital, 11527 Athens, Greece; 3 Athens, Greece; 4 Drama, Greece

**Keywords:** Asthma, Asthma treatment, Adherence, Quality of life, Budesonide/formoterol

## Abstract

**Background:**

Asthma is a chronic inflammatory disease of the airways that causes recurring episodes of wheezing, breathlessness, chest tightness and coughing. Inhaled drugs on a daily basis are the cornerstone of asthma treatment, therefore, patient adherence is very important.

**Methods:**

We performed a multicenter, open, non-interventional, observational, prospective study of 716 adult patients diagnosed with asthma receiving FDC (Fixed-dose combination) budesonide/formoterol via the Elpenhaler device. We assessed the adherence to treatment at 3 and 6 months (based on the MMAS-8: 8-item Morisky Medication Adherence Scale), the quality of life and change in forced expiratory volume in 1 s (FEV1) from baseline to follow-up.

**Results:**

Approximately 80% of the patients showed medium to high adherence throughout the study. The mean (SD) MMAS-8 score at 6 months was 6.85 (1.54) and we observed a statistically significant shift of patients from the low adherence group to the high adherence group at 6 months. Moreover, after 6 months of treatment with FDC budesonide/formoterol, we observed an increase in the patients’ quality of life that as expressed by a change 2.01 (95%CI 1.93–2.10) units in Mini AQLQ (*p* < 0.0001) that was more pronounced in the high adherence group. The same trend was also observed in terms of spirometry (mean FEV1 2.58 L (0.85) at the end of the study, increased by 220 mL from baseline) with a higher improvement in the medium and high adherence groups.

**Conclusions:**

Treatment with FDC of budesonide/formoterol via the Elpenhaler device was associated with improvement in asthma-related quality of life and lung function over 6 months that were more prominent in patients with higher adherence.

*Trial registration*: 2017-HAL-EL-74 (ClinicalTrials.gov Identifier: NCT03300076).

## Introduction

Asthma is a chronic, common and heterogeneous respiratory disease, characterized by diffuse airway inflammation. Its prevalence varies between 1–18% across different countries; the respective prevalence in Greece is estimated around 8.6% [1]. Patients often complain about recurring episodes of wheezing, shortness of breath, chest tightness and cough of variable intensity over time, together with variable expiratory airflow limitation. The majority of people diagnosed with asthma achieve good or very good control of their disease and are able to live a normal life, punctuated only by the need to take regular inhaled medication and by occasional exacerbations. Based on the triad of asthma control, severity and exacerbations, the patient’s treatment is reevaluated and fine-tuned based on a 5-step treatment scale [2].

Asthma control refers to the extent that asthma symptoms are reported by the patient. Therefore, asthma control reflects the same domains targeted by asthma management, i.e. symptom control and future risk of adverse outcomes. To this end, several scores have been proposed in the literature such as Asthma APGAR, ACQ (Asthma Control Questionnaire) and ACT (Asthma Control Test) to name a few. Pulmonary function tests, and spirometry in particular, constitute an objective means of assessing the patient’s status, either compared to predicted values or by measuring deviations from the patient’s baseline or ‘personal best’. Even though spirometry does not always correlate strongly with asthma symptoms, it may offer quantitative information for the periodic assessment of asthma patients.

The pharmacological treatment of asthma includes controller/maintenance medication, i.e. inhaled corticosteroids (ICS), with or without long-acting β2-agonists (LABA), and reliever medication taken as required to relieve symptoms, such as short-acting β2- agonists (SABA) or ICS-formoterol combinations. Treatment regimens with ICS-formoterol can be used both as controller and reliever medication, as well as as-needed medication in milder asthma, based on the GINA 2020 recommendations [2–4]. Moreover, the ICS-formoterol combination constitutes the cornerstone of asthma treatment from step 1 up to step 4 of the GINA 2020 recommendations. Therefore, choosing the appropriate controller and/or reliever medication is of great significance in the asthma treatment and the asthma management, overall.

Besides choosing the right treatment for each patient, conforming to the treatment (i.e. medication adherence) is also necessary. Medication adherence is a term of great importance that affects nearly every asthma aspect, such as symptom control, treatment decision and escalation, severity assessment and asthma prognosis. Medication adherence is also important for differentiating between severe asthma and difficult-to-treat asthma. Moreover, poor adherence is an independent risk factor for predicting future asthma exacerbations and persistent airflow limitation. Medication adherence is affected by a wide array of factors, e.g. multiple devices, difficult treatment plan (multiple times per day), forgetfulness, cost, concerns about side-effects [5]. The device in particular has been found to play an important role in the correct drug administration and the patient’s adherence to treatment [6, 7]. Even though there is already a wide range of devices in the market, the development of novel inhaler devices is a necessity in order to (i) achieve targeted drug delivery and (ii) improve the patients’ condition by facilitating adherence [8].

The routine identification of non-adherent patients followed by targeted interventions can lead to increased adherence. Several methods for adherence assessment are available in the literature each with a set of advantages and disadvantages [9]. These include subjective monitoring tools e.g. physician assessment of adherence or self-report questionnaires, as well as objective monitoring approaches, such as: prescription data, dose counters, directly observed therapy, etc. [10, 11]. The current gold standard for assessing adherence is electronic monitoring which provides a load of inhaler related information for further analysis. Nevertheless, electronic monitoring devices are often expensive and do not assess the inhaler technique [10, 12].

Another important aspect in asthma management is the quality of life perceived by the patient. As noted earlier, in some cases there is discordance between spirometry and symptomatology which reflects the overall quality of life. For this purpose, clinical scores have been proposed to quantify the life quality of patients diagnosed with asthma of variable severity [13]. AQLQ (Asthma Quality of Life Questionnaire) as well as its short version Mini AQLQ [14] are two most widely used scores for measuring asthma life quality.

In the current study, we have considered a large set of approximately 700 patients diagnosed with asthma of variable severity. The enrolled patients received treatment with fixed dose combinations (FDC) of budesonide/formoterol via the Elpenhaler device, according to usual clinical practice and were reevaluated at 3 and 6 months to assess adherence to treatment, quality of life and change in spirometry.

## Materials and methods

### Study design

This is an open label, multi-center, non-interventional, non-comparative, observational, prospective study (NCT03300076) of adult patients diagnosed with asthma of variable severity. In total, 716 patients were enrolled and followed-up for up to 6 months in 53 institutions and private practices throughout Greece, commencing from February 2018 and up to June 2018. The study was performed in accordance with the principles of the Declaration of Helsinki and was approved by the Ethics Committees of the Sotiria Chest Disease Hospital and the General Hospital of Chalkida. Written informed consent was obtained from all participants. Patients were treated with budesonide/formoterol FDC (Pulmoton Elpenhaler), according to routine clinical practice. The majority of the enrolled patients were treatment naive, and the ones already on treatment were either not satisfactorily controlled with ICS (Inhaled Corticosteroids) and on demand use of SABAs (Short Acting Beta Agonists), or were patients already satisfactorily controlled with both ICS and LABA, though administered with 2 different devices. We excluded patients meeting any of the following criteria: diagnosis of Chronic Obstructive Pulmonary Disease (COPD) at any stage, use of any fixed combination of ICS/LABA at least 1 month prior to study initiation, and/or prior use of systemic corticosteroids within 3 months from study initiation. Patients with a history of improper use of inhaled therapies or failing to comply with the study procedures, were also excluded. After study initiation, patients used a fixed dose arrangement of the inhaled treatment as instructed by the treating physician.

The primary objective of this study was to evaluate the adherence to treatment with FDC budesonide/formoterol at 3 and 6 months after treatment initiation. Adherence to treatment was assessed at 3 and 6 months since treatment initiation based on the 8-item Morisky Medication Adherence Scale (MMAS-8) [15–19], which has been elsewhere used primarily in chronic diseases, e.g. type II diabetes [20], hypertension [21], etc. This self-reported scale contains 7 items answered with a yes or no and 1 item with a 5-point Likert scale, with scores ranging from 0 to 8. The respective MMAS-8 scores were trichotomized into the following 3 levels of adherence: high adherence (HA, score = 8), medium adherence (MA, 6 ≤ score < 8), and low adherence (LA, score < 6).

Quality of life (QoL) was assessed using the validated Greek version Mini Asthma Quality of life Questionnaire (Mini AQLQ) [22] at baseline, 3 and 6 months, depicting the impact of asthma in the patient’s QoL. This 15-item questionnaire consists of 4 domains: (i) symptoms, (ii) environment, (iii) emotions, and (iv) activities, and covers a 2 week period. Scores range from 1–7 (lower is worse).

Spirometry was also performed in the same visits, using the ERS/ATS guidelines [23] and forced expiratory volume in 1 s (FEV1), forced vital capacity (FVC) and their ratio (FEV1/FVC) were recorded. Moreover, asthma related exacerbations after 3 and 6 months since treatment initiation were recorded for each patient. In terms of safety, adverse events were reported throughout the study; the most current version of Medical Dictionary for Regulatory Activities (MedDRA v21.1) was used for the medical coding of the recorded adverse events.

### Statistical analysis

Descriptive statistical analysis was performed on all patients in terms of patient demographics (sex, age, weight, height, smoking status, etc.) and spirometric data. Other characteristics such as history of asthma and prior medications were also summarized. Continuous variables were summarized with the use of descriptive statistical measures [mean value, standard deviation (SD), median, IQR] and categorical variables were displayed as frequency tables (N, %). Association between categorical variables was presented by contingency tables and assessed using Chi-square test or Fisher exact test, when appropriate. Furthermore, in order to examine possible differences between continuous variables, paired t-test for related samples was applied. All the statistical tests were two-sided and were performed at a 0.05 significance level. Analysis was performed on the basis of non-missing information and no imputation methods were applied.

For the primary objective of the study, i.e. adherence to treatment with Pulmoton Elpenhaler, the MMAS-8 scale was used. Continuous scale score (0–8) was descriptively summarized at 3 and 6 months and change in score between study visits was evaluated by paired t-test. The MMAS-8 score was also categorized into “high” (score = 8), medium (score = 6–7), and “low adherence” (score = 0–5) and summarized by absolute and relative frequencies (N, %). According to Muntuner et al. [24], the minimal detectable change for MMAS-8 score is 1.98.

Quality of life was assessed with the Mini AQLQ at 0, 3 and 6 months of the study. Individual scores per question, total score and 4 domain scores (symptoms, environment, emotions, activities) were descriptively summarized and changes in the total score at 3 and 6 months from baseline were further assessed by paired t-test. Efficacy of the study drug was assessed in terms of spirometry results at 0, 3 and 6 months of the study. Additionally, changes in the Mini AQLQ questionnaire between 3 and 6 months have been evaluated with analyses of covariance (ANCOVA), using baseline values as covariates. All spirometry data (FEV1 measured in L, FEV1% predicted, FVC measured in L, FVC % predicted, and FEV1/FVC ratio) were summarized by mean, SD, median and IQR and changes in key spirometry data were further assessed by paired t-test. The minimal clinically important difference in patients with asthma has not been rigorously established in asthma, but a report from the US NIH suggests that changes of 100–200 mL are likely to be clinically important [25]. Statistical analysis was performed by means of IBM-SPSS v24.0 statistical software.

## Results

### Baseline demographics and clinical characteristics

The study included 53 sites in Greece, which overall enrolled 716 patients. There were no patients excluded from the analysis, thus the study population analyzed was equal to 716 patients. The follow-up visits were performed at 3 and 6 months, where 684 (95.5%) and 666 (93%) patients completed each visit, respectively. Otherwise, there were no missing values, therefore, no imputation methods were applied. Amongst the 716 patients included in the study, 455 were women (63.5%), and 99.6% were of white race. Mean age was 52 years with 39% of the patients being 40–59 years. The median body mass index (BMI) of patients in the study was approximately 28, a rate indicating that the majority of patients had increased body weight. In addition, approximately half of the study patients were of higher education (Table 1).

**Table 1:** Demographic and clinical characteristics of patients at baseline

	(Ν, %)
Age-yr	N = 716
Mean (SD)	52.3 (16.5)
Age group-no. (%)	N = 716
< 40	169 (23.6%)
40–59	279 (39%)
60–79	248 (34.6%)
≥ 80	20 (2.8%)
Gender-no (%)	N = 716
Male	261 (36.5%)
Female	455 (63.5%)
BMI-kg/m^2^	N = 716
Mean (SD)	28.6 (5.8)
Education-no. (%)	N = 716
Basic (6 years of education)	149 (20.8%)
Higher (12 years of education)	360 (50.3%)
University (University degree)	207 (28.9%)
Smoking status-no. (%)	N = 716
Non-smoker	423 (59.1%)
Ex-smoker	144 (20.1%)
Current smoker	149 (20.8%)

In this study, 59.1% of the patients were non-smokers. 20.1% were ex-smokers with median pack-years equal to 20, while 149 patients (20.8%) were active smokers with median pack-years equal to 15. 42 out of the 149 smokers (28.2%) stopped smoking during the study, decreasing the number of smokers to 107 (15%).

**Fig. 1:**
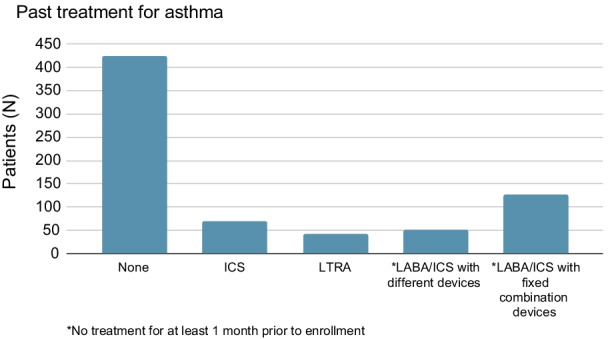
Past treatment for asthma (until the day of enrollment)

The asthma treatment of the enrolled patients prior to the study is depicted in Fig. 1; 59.4% of the patients were treatment-naive, while 291 patients (40.6%) had at least one past asthma treatment, with 180 patients having received 2 treatments. The most common past asthma treatment seemed to be a fixed combination of inhaled corticosteroids and LABA (128 patients). Note also that approximately 15.4% of the study patients had previously received concomitant asthma-related medications with 68 out of them (61.8%) having received leukotriene receptor antagonists (LTRA); 57.5% of the patients had not received any on-demand treatment until the day of enrollment.

### Medication adherence

The mean (SD) MMAS-8 score at month 3 and 6 was 6.59 (1.74) and 6.85 (1.54) respectively. We observed a statistically significant increase of 0.20 (95%CI 0.08–0.32) units (*p* = 0.001) in MMAS-8 score at 6 months from Visit 1 (Month 3). The MMAS-8 scores for each patient were also categorized into “HA = high adherence” (score = 8), “MA = medium adherence” (score = 6–7.99), and “LA = low adherence” (score = 0–5.99). The specific proportions of patients in the 3 categories are presented in Table 2.

**Table 2:** Adherence of asthma patients to treatment, as assessed by the MMAS-8 scale at 3 and 6 months

MMAS-8 score	Visit 1 (month 3) *N* = 684	Visit 2 (month 6) *N* = 666
High adherence (score = 8)	274 (40.1)	315 (47.3)
Medium adherence (score = 6–7.99)	227 (33.2)	216 (32.4)
Low adherence (score = 0–5.99)	183 (26.8)	135 (20.3)

**Fig. 2:**
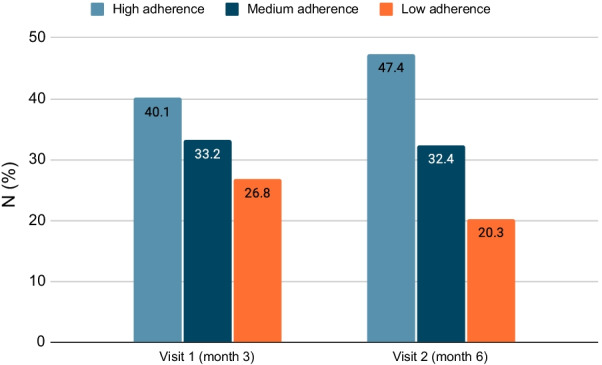
Adherence to treatment, as assessed by MMAS-8 scale at 3 and 6 months

As shown in Fig. 2, approximately 80% of the patients showed medium to high adherence (MMAS-8 score ≥ 6) throughout the study, however the patients in this category differed significantly between 3 and 6 months (McNemar's test *p* < 0.0001). More specifically:

- There were 91 out of 183 patients (49.7%) changing from low to medium (55 patients) and high adherence (36 patients).
- There were 71 out of 227 patients (31.3%) changing from medium to high adherence.

[The Morisky Widget, MMAS-8. MMAS-4 are protected by US and International Trademark and Copyright laws. Permission for use is required. A license agreement is available from: MMAS Research LLC 14,725 NE 20th St. Bellevue WA 98007].

### Quality of life

**Fig. 3:**
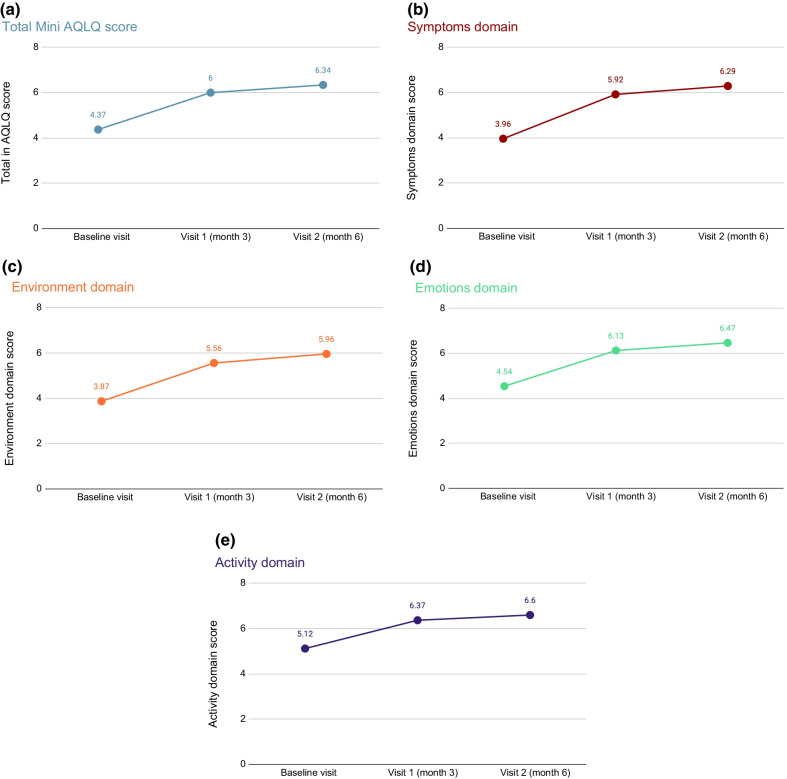
**a** Total Mini AQLQ score. **b** Symptoms domain score from the Mini AQLQ score. **c** Environment domain score from the Mini AQLQ score. **d** Emotions domain score from the Mini AQLQ score. **e** Activities domain score from the Mini AQLQ score

The total Mini AQLQ score and individual domain scores are presented in Figs. 3a–e. Specifically, Fig. 3a depicts the total Mini AQLQ score. The mean (SD) total score was 4.37 (1.13), 6.00 (0.77) and 6.34 (0.62) at baseline, 3 months and 6 months respectively and the change from baseline was 1.66 (95%CI 1.58, 1.74) and 2.01 (95%CI 1.93–2.10) units at 3 and 6 months, respectively (*p* < 0.0001 for both comparisons), indicating that the quality of life of the patients was improved overall at the end of the study.

Figures 3b–e show the individual scores for the 4 domains (symptoms, environment, emotions and activities, respectively). Specifically, for the Symptoms domain score the change from baseline was 1.96 (95%CI 1.87, 2.05) and 2.36 (95%CI 2.26, 2.45) units at 3 and 6 months; for the Activities domain score the change from baseline was 1.25 (95%CI 1.16, 1.35) and 1.51 (95%CI 1.41, 1.62) units at 3 and 6 months, respectively; for the Emotions score the change was 1.63 (95%CI 1.53, 1.73) and 1.98 (95%CI 1.87, 2.09) units; and as for the Environment domain score the change from baseline was 1.73 (95%CI 1.64, 1.82) and 2.15 (95%CI 2.05, 2.24) units at 3 and 6 months. It should be noted that for all aforementioned comparisons *p* < 0.0001. The highest score amongst all domains was the activities domain score which was the highest throughout the study, with a median score at 6 months equal to 6.75. Nevertheless, the median score of the aforementioned domain at the end of the study was 6, significantly increased by 2.15 units from baseline (*p* < 0.0001).

**Fig. 4:**
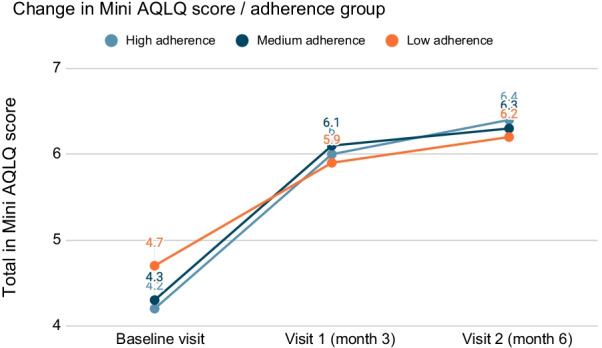
Change in Mini AQLQ score in the three consecutive visits, per adherence group

Quality of life of the study patients was also evaluated by the Mini AQLQ at 3 and 6 months per treatment adherence group during the same months (data not shown). Overall, almost all mean scores per question were higher or equal at 3 months for the patients with medium adherence to study treatment. Social activities were scored the highest amongst all questionnaire items for every treatment adherence group. Regarding the 6-month assessment of the Mini AQLQ, all mean scores were slightly higher than in 3 months, thus, the quality of life of patients with low, medium and high adherence was improved. Overall, high adherence patients showed greater improvement in their quality of life compared to low and medium adherence groups, having the highest scores per domain and in total across the three visits (Fig. 4).

**Fig. 5:**
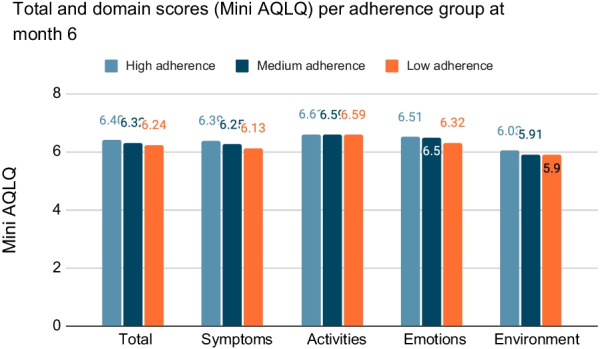
Mini AQLQ scores in total as well as in each domain, per adherence group

Moreover, we have assessed the difference of Mini AQLQ scores in total as well as in each domain, per adherence group (Fig. 5). We observed that the high adherence group had the greatest improvement in their quality of life compared to medium and low adherence groups, as shown in the Mini AQLQ scores per each domain (symptoms, activities, emotions, and environment) and in total.

### Spirometric results

The effectiveness of the FDC budesonide/formoterol treatment via the Elpenhaler device was evaluated by spirometry data at baseline, 3 months and 6 months. The mean (SD) FEV1 was 2.58 L (0.85) at the end of the study, increased by 220 mL from baseline. More specifically, for the low adherence group the mean (SD) FEV1 in L across all 3 visits was 2.42 (0.87) L, 2.68 (0.89) L and 2.59 (1.09) L; for the medium adherence group the mean (SD) FEV1 was 2.32 (0.91) L, 2.57 (0.90) L, 2.57 (1.09) L; and for the high adherence group the respective values were 2.35 (0.88) L, 2.52 (0.91) L, and 2.57 (0.86) L.

**Fig. 6:**
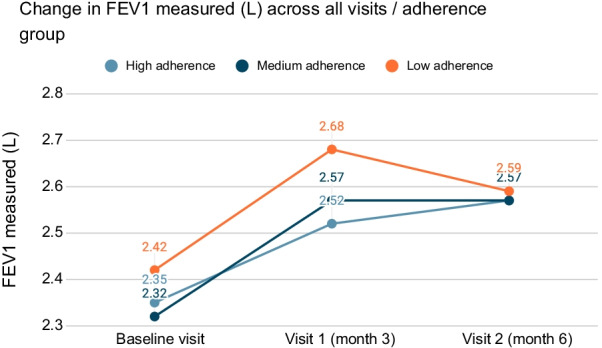
Change on FEV1 measured (L) across all visits, per adherence group

We examined the changes in spirometry for each adherence group, based on MMAS-8. We observed a slight drop in FEV1 measured from 3 to 6 months only for the low adherence patients. In the medium adherence group the mean FEV1 measured increased from baseline to 3 month visit and remained unchanged in the 6 month visit. As for the high adherence group we observe a gradual increase from baseline to 3 month visit and subsequently to the 6 month visit. Moreover, change from baseline at 6 months was lower in low adherence patients than in medium and high adherence patients (170 mL versus 250 mL and 220 mL, respectively). The minimal clinically important difference in patients with asthma according to a US NIH report suggests that changes of 100–200 mL are likely to be clinically important [25]. The fact that there was such an improvement in FEV1 in all groups, but the mean improvement was higher in the medium and high adherence groups, suggests that all patients received an effective treatment, however, the improvement in lung function was more prominent in those with better adherence. The change on FEV1 measured in L across all visits per adherence group is shown in Fig. 6.

### Exacerbations and safety

Regarding asthma exacerbations, 30 (4.4%) and 26 (3.9%) of the patients had at least one asthma-related exacerbation during 3 and 6 months of the study respectively. Of the 30 patients who experienced an exacerbation during the first 3 months of the study, 18 had their treatment modified and 1 patient discontinued. Moreover, 13 (43.3%) and 11 patients (42.3%) reported that they had received oral corticosteroids for their exacerbations at Visits 1 and 2 respectively. None of the patients was hospitalized due to an exacerbation throughout the study period.

The safety of the treatment under consideration was assessed in terms of adverse events and severe adverse events. Overall, only 4 patients (0.6%) had at least one adverse event throughout the study period; none of the patients had a severe adverse event. Two patients (0.3%) discontinued the initial treatment permanently due to an adverse event, while 3 patients (0.4%) had a dose decrease. The most common adverse event was tachycardia, which occurred in 2 patients.

## Discussion

In the current 6-month observational study, we have recruited approximately 700 adult asthmatic patients that were treated with FDC of budesonide/formoterol via the Elpenhaler device, according to usual clinical practice. The majority of patients reported medium to high adherence at 3 months with an increase in adherence at 6 months. We observed an improvement in quality of life (as evaluated by the Mini AQLQ questionnaire) and in lung function (as expressed by FEV1 in liters) and these improvements were more evident in the high adherence patients. The safety profile of the FDC of budesonide/formoterol via the Elpenhaler device was acceptable and in accordance with previous reports.

The main finding of this study is that patients with medium or high adherence to the inhaled medication under consideration reported higher quality of life even after a relatively small period of time, i.e. 3 or 6 months of treatment. The finding applies to all enrolled patients indifferent to the severity of asthma. Focusing on patient subsets based on asthma severity in order to assess the impact of inhaled medication in each set falls outside the scope of this study, but could be an interesting future prospect. Moreover, we observed that medium and high patient adherence to treatment had a positive impact on spirometric results, especially in terms of FEV1, which is in accordance with the literature. Therefore, our results further support the observation that adherence to treatment is central in the overall course of asthma, and should be stressed by the physicians in every follow-up visit, as proposed by current recommendations [2]. Nevertheless, the design of the study does not allow to conjecture safely about the effectiveness of the budesonide/formoterol combination delivered via the Elpenhaler device.

In patients diagnosed with asthma, the choice of treatment is largely decided based on recent guidelines. Subsequently, the main responsibilities of the treating physician are: (i) identify the specific inhalation device that will satisfy the patient the most and (ii) achieve and maintain adherence to the device over the follow-up. The former point has been studied in a previous study [26] where several inhalation devices were prospectively evaluated in Greek patients with COPD and asthma. The patients’ satisfaction was assessed using a standardized questionnaire. Among patients with asthma, the inhalation device used in the current study, i.e. the Elpenhaler, presented significantly higher satisfaction rates compared to the other devices. The patient’s satisfaction with their inhaler device has been linked with adherence to treatment, disease control, disease clinical course and clinical outcomes [27]. Therefore, the results of high adherence that improves over time in the present observational study may be partly attributed to the appropriate use of the Elpenhaler device. To this end, it is also noteworthy that the Elpenhaler device, when compared with some other frequently used inhalation devices, exhibited the lowest error rates for critical errors in the inhalation maneuver [28]. Another important finding of that study is that physical demonstration of correct inhalation maneuvers prior to first administration leads to higher percentage of adequate use and minimization of critical errors [28]. Our results are in line with previously published data suggesting Elpenhaler as a ‘‘self-improvement’’ device [29]. Based on the results reported in the same study [29], the Elpenhaler device was rated best in 7 out of 10 questions of the FSI-10 (Feeling of Satisfaction with Inhaler) questionnaire in asthma patients, and in 8 out of 10 questions in COPD patients. The features praised about the Elpenhaler device were: ease in learning and keep using the inhaler, verification of dose delivery, as well as factors related to size, weight, cleanliness and unobtrusiveness in everyday activities. In the recently published BOREAS study [30], the authors present some important clinical insights regarding the real-life effectiveness of FDC budesonide/formoterol via the Elpenhaler device in 1230 asthmatic patients. Specifically, the authors reported significant improvements in asthma control (based on ACQ-7) and quality of life (Mini AQLQ) at 3 months that were sustained after 6 months.

Another factor that should be taken into consideration before choosing an inhaler device is carbon footprint. Even though it is often neglected during clinical practice, it has recently attracted considerable attention, and nation-wide studies have been conducted for this purpose [31]. Dry-powder inhalers (DPIs) compared to metered dose inhalers (MDIs) have a considerably lower carbon footprint, and the difference is mainly related to the use of the inhaler as well as the disposal of the device. Therefore, Elpenhaler being a DPI offers an appealing solution from an environmental point of view, especially compared to an MDI device. Nevertheless, choosing an inhaler device or switching between devices is an important and multifactorial decision that should be based on thorough clinical assessment coupled with patient education and training.

The combination of ICS and LABA has been studied in large cohorts for the management of asthma with results that have established this treatment option as the basis of the management of a great proportion of patients with asthma [32]; its effectiveness can be partly attributed to the synergy observed between ICS and LABA at molecular level that has been reported via multiple mechanisms and pathways [33].

Further focusing on the impact of specific devices delivering budesonide/formoterol, Syk et al.[34] have studied the effectiveness of switching between two popular inhalers. This study signifies the importance of the device for inhaled therapies, specifically for the substances under consideration in this article, i.e. budesonide/formoterol. The authors report a statistically significant improvement in asthma control as well as life quality after 6 months, as measured by ACT and Mini AQLQ, respectively. Based on the latest version of the GINA guidelines [35], ICS-formoterol represents a preferred option for the management of patients with asthma across all steps. It can be used both as a controller and a reliever with variable dosing based on asthma severity and treatment step. Budesonide/Formoterol via the Elpenhaler device that is studied herein, qualifies as a single inhaler maintenance and reliever therapy (SMART), that is the preferred treatment in GINA guidelines [35], because using ICS-formoterol as reliever reduces the risk of severe exacerbations compared with regimens with SABA as a reliever [36]. Moreover, having the two substances in a single device is more practical for daily administration and facilitates better adherence and compliance.

In the current real-life study, by setting relatively broad inclusion criteria we have achieved a satisfactory sample size with considerable heterogeneity consisting of patients with asthma of variable severity, coming both from the Hospital setting as well as from several private practices. One the other hand, our study has some limitations. Since we enrolled patients from a single country, generalizations in terms of epidemiology should be considered with caution. As in most studies, the Hawthorne effect is also evident, albeit it is difficult to evaluate its contribution. Moreover, due the study design, being one-arm in particular, makes it difficult to assess and quantify the impact of treatment and adherence to lung function; this is partly compensated by the selection of both subjective (MMAS-8, Mini AQLQ) and objective (lung function) metrics for the study purpose. However, the fact that we observed corresponding improvements in both subjective and objective measures further supports the real-life effectiveness of the studied ICS/LABA combination.

## Conclusions

In this study of 716 asthmatic patients, treatment with FDC of budesonide/formoterol via the Elpenhaler device was associated with medium to high adherence in the majority of patients (~ 80%), with improvement in asthma-related quality of life and lung function over 6 months. These improvements were more prominent in patients with higher adherence.

## Data Availability

The datasets used and/or analysed during the current study are available from the corresponding author on reasonable request.
