# Correction to: Adherence and quality of life assessment in patients with asthma treatment with budesonide/formoterol via the Elpenhaler device: the COMPLETE study

**DOI:** 10.1186/s12890-022-02117-5

**Published:** 2022-08-23

**Authors:** Konstantinos P. Exarchos, Nikoletta Rovina, George Krommidas, Dimitrios Latsios, Athena Gogali, Konstantinos Kostikas

**Affiliations:** 1 Respiratory Medicine Department, University of Ioannina School of Medicine, Ioannina, Greece; 2 1St Department of Respiratory Medicine, Medical School, National and Kapodistrian University of Athens and “Sotiria” Chest Disease Hospital, 11527 Athens, Greece; 3 Athens, Greece; 4 Drama, Greece

## Correction to: BMC Pulmonary Medicine (2022) 22:254 10.1186/s12890-022-02049-0

Following publication of the article [1], the authors flagged that the following acknowledgement text had been erroneously omitted: "The MMAS-8 Scale, content, name, and trademarks are protected by US copyright and trademark laws. Permission for use of the scale and its coding is required. A license agreement is available from MMAR, LLC., www.moriskyscale.com." In addition, they highlighted that two references had been left out in error.

The original article has since been updated with the acknowledgement text and the two references (references 20 and 21). The authors thank you for reading this erratum and apologize for any inconvenience caused.
